# Characteristics of admissions and variations in the use of basic investigations, treatments and outcomes in Kenyan hospitals within a new Clinical Information Network

**DOI:** 10.1136/archdischild-2015-309269

**Published:** 2015-12-10

**Authors:** Philip Ayieko, Morris Ogero, Boniface Makone, Thomas Julius, George Mbevi, Wycliffe Nyachiro, Rachel Nyamai, Fred Were, David Githanga, Grace Irimu, Mike English

**Affiliations:** 1 Kenya Medical Research Institute/Wellcome Trust Research Programme, Nairobi, Kenya; 2 Division of Maternal, Newborn, Child and Adolescent Health, Ministry of Health, Nairobi, Kenya; 3 School of Medicine, University of Nairobi, Nairobi, Kenya; 4 Kenya Paediatric Association, Nairobi, Kenya; 5 Department of Paediatrics and Child Health, University of Nairobi, Nairobi, Kenya; 6 Nuffield Department of Medicine, Oxford University, UK

**Keywords:** Epidemiology, Tropical Paediatrics, Health services research, Mortality, Paediatric Practice

## Abstract

**Background:**

Lack of detailed information about hospital activities, processes and outcomes hampers planning, performance monitoring and improvement in low-income countries (LIC). Clinical networks offer one means to advance methods for data collection and use, informing wider health system development in time, but are rare in LIC. We report baseline data from a new Clinical Information Network (CIN) in Kenya seeking to promote data-informed improvement and learning.

**Methods:**

Data from 13 hospitals engaged in the Kenyan CIN between April 2014 and March 2015 were captured from medical and laboratory records. We use these data to characterise clinical care and outcomes of hospital admission.

**Results:**

Data were available for a total of 30 042 children aged between 2 months and 15 years. Malaria (in five hospitals), pneumonia and diarrhoea/dehydration (all hospitals) accounted for the majority of diagnoses and comorbidity was found in 17 710 (59%) patients. Overall, 1808 deaths (6%) occurred (range per hospital 2.5%–11.1%) with 1037 deaths (57.4%) occurring by day 2 of admission (range 41%–67.8%). While malaria investigations are commonly done, clinical health workers rarely investigate for other possible causes of fever, test for blood glucose in severe illness or ascertain HIV status of admissions. Adherence to clinical guideline-recommended treatment for malaria, pneumonia, meningitis and acute severe malnutrition varied widely across hospitals.

**Conclusion:**

Developing clinical networks is feasible with appropriate support. Early data demonstrate that hospital mortality remains high in Kenya, that resources to investigate severe illness are limited, that care provided and outcomes vary widely and that adoption of effective interventions remains slow. Findings suggest considerable scope for improving care within and across sites.

**What is already known on this topic**

- Quality of paediatric care in county (district) hospitals in low-income countries can be poor.
- Quality improvement initiatives are hampered by widespread deficiencies in data collection and use.
- Clinical networks provide a platform for effective implementation of quality improvement in high-income countries but networks spanning non-specialist facilities delivering routine care appear rare in low-income countries.

**What this study adds**

- Developing clinical networks spanning general hospitals providing paediatric care in low-income countries is feasible with appropriate support.
- Initial data from a new clinical network show that quality of care in county (district) hospitals varies across hospitals, disease conditions and processes of care suggesting that there is significant scope for quality improvement.
- The reported experience indicates a possible role of clinical networks in developing better health information systems that in turn could play an important cross-cutting role in supporting local improvement efforts, benchmarking and tracking adoption of interventions.

## Background

Global and national attention is beginning to turn to universal coverage with quality healthcare.^1^ To achieve this will require the development of health systems in low-income countries (LIC) that equitably deliver effective interventions at scale, and careful decisions about how to improve existing care must be made.^2^ ^3^ To inform such decisions, we require information on major causes of morbidity and mortality, what interventions are being delivered, to whom and with what outcomes. Marked variability in care and outcomes may demonstrate the need for efforts to support implementation of key interventions or wider system strengthening. So do we have such information? At present, episodic surveys or specific infectious disease surveillance mechanisms provide limited information. Data may also be available from routine district health information systems.^4^ However, these systems often cover a limited number of indicators and data quality, even outcome data for inpatients, may be poor.^5^

To foster better generation and ultimately better use of information, we have established a clinical information network (CIN) focused on hospitals’ inpatient paediatric units in Kenya. Partners in this effort include the Ministry of Health, the Kenya Paediatric Association, the hospitals and the research team. The approach borrows from ideas of networks as ‘a grouping that aims to improve clinical care and service delivery using a collegial approach to identify and implement a range of (improvement) strategies’.^6^ Such clinical networks have been a feature of efforts to improve care in high-income and middle-income countries.^7^ ^8^ In the first phase of network activities we aimed to address the question what do hospitals currently do? Specifically we wished to characterise paediatric admissions and explore the use of recommended treatments, basic diagnostics and technologies. From this starting point, the aim of the network is to examine ways of improving care and outcomes.

## Methods

### Hospital selection

Kenya devolved healthcare provision to 47 county administrations in 2013. In establishing CIN, 12 counties were identified purposefully with the Ministry of Health to represent two main groupings based on high or low/very low malaria prevalence. Public hospitals within these counties were estimated to have at least 1000 paediatric admissions per year and excluding tertiary-level facilities were purposefully selected to ensure the feasibility of the project. Two hospitals from the largest county serving a population of over 3 million were included and in one county two adjacent facilities located in the same town, one a smaller community hospital, were included as this was logistically straightforward. The nature and purpose of the network was explained and all 14 hospitals agreed to take part. However, this report presents data from the 13 county (district) hospitals that are first referral centres as the smaller community hospital has a somewhat different function.

### The Clinical Information Network

The paediatrician, the nurse in charge of the paediatric unit and the senior health records information officer, as key partners, participated in an introductory workshop to explain the approach to and purpose of data collection. These introductory meetings were also used to remind hospitals about the national guidelines for common conditions that have been disseminated for some time.^9^ Importantly, the network does not provide any material or financial resources to the hospitals or individuals but, through 4 monthly meetings, telephone calls and provision of information back to hospitals in the form of two monthly reports, CIN has tried to build a sense of partnership across the hospitals.

### Data collection and analysis

A detailed description of the methods of data collection and analysis are reported elsewhere.^10^ In brief, the network encouraged hospitals to implement two data collection tools (a paediatric admission record and discharge form) and also provided support for one additional clerical assistant in each hospital to collect data from the medical records and laboratory reports. Data collection was conducted as soon as possible after discharge through abstracting data from inpatient paper records into a non-propriety electronic tool, REDCap. Data required for the national reporting system were collected for each paediatric admission in each hospital and more comprehensive data on disease-specific care processes including investigations and treatment were collected in all acute medical admissions (excluding neonates) in 11 low-to-moderate workload hospitals and on a random subset of similar medical admissions in three high workload hospitals.

The first hospitals joined the network in October 2013 the last in February 2014. Data for children older than 1 month and less than 15 years, admitted between 1 April 2014 and 31 March 2015, were analysed. The frequency distributions of admission diagnoses were examined stratified by age and malaria prevalence. Diagnoses used in all analyses were based on ICD-10 coding supplemented by additional coding to capture disease severity classifications recommended in national and WHO guidelines.^11^ ^12^ Data on children admitted with surgical diagnoses are excluded from analysis as they are typically admitted to surgical wards outside the scope of the network. The disease burden analysis is based on diagnostic episodes and thus allowed for more than one diagnosis per child (also meaning the number of disease episodes requiring treatment is greater than the number of admissions).

For children admitted with meningitis or severe acute malnutrition, we assumed these to be the primary cause of death in the face of any other comorbidity (if both diagnoses were made, we assumed meningitis was the cause of death).^13^ We did not attempt to assign a single cause of death after excluding meningitis and severe acute malnutrition deaths. All remaining deaths of medical causes are presented using Venn diagrams to overcome the problem of double-counting deaths in children with multiple diagnosis. A separate analysis including all deaths regardless of diagnosis was conducted to describe the distribution of paediatric deaths by day during the first week of admission. Time of admission and death are rarely documented so the approach based on dates slightly overestimates survival time.

The analysis of treatment and investigation practices contrasts care with national paediatric clinical guidelines that are evidence-based and largely reflect WHO guidance.^11^ ^12^ We present proportions of cases with orders and reported results for essential laboratory investigations recommended in national guidelines for all admissions (HIV testing), for severely ill children (blood glucose) or for specific conditions (malaria, meningitis and severe pallor). There is no specific guidance on use of additional investigations but we were interested in the proportion of children with fever who had a blood culture or urine testing (including dipstix, microbiology and culture) performed and children with pneumonia who had a chest X-ray ordered. Finally, we report percentages of children who are prescribed guideline recommended drugs for their specific condition, and we also conduct dosing analysis for gentamicin (a drug with known toxicity).

## Results

### Participating hospitals and patients

Characteristics of the 13 hospitals are provided in table 1. After exclusion of neonates (n=203) and some children with surgical diagnoses (n=788) admitted to medical wards, data for 30 042 medical admissions were analysed. Five hospitals were located in high malaria prevalence areas and contributed 45% of all observations. The median age (IQR) at admission ranged quite substantially from 12 (7–24) to 30 (13–60) months across hospitals, but hospital regulations on age limits for admission into wards also varied, with 7 out of 13 hospitals admitting children up to 12 years, and 3/13 and 2/13 hospitals admitting children to 13 and 15 years, respectively.

**Table 1:** Characteristics of hospitals participating in Clinical Information Network and paediatric admissions

	Hospitals												
	H1	H2	H3	H4	H5	H6	H7	H8	H9	H10	H11	H12	H13
Hospital or catchment population characteristics													
Ward bed capacity	32	63	35	38	29	67	29	38	35	42	41	32	21
Admission age range	1 month–12 years	1 day–12 years	1 day–15 years	1 day –12 years	1 day–12 years	1 day–12 years	1 day–13 years	1 day–13 years	1 day–12 years	1 day–12 years	1 day–12 years	1 day–15 years	1 month–12 years
Percentage living in extreme poverty	65	41	51	49	25	26	45	49	56	21	21	31	40
Adult HIV prevalence (%)	7.1	3.7	5.6	4.4	4	4.4	18.7	7.2	4.7	8.6	8.6	4.4	6
Malaria prevalence	High	Low	High	Low	Low	Low	High	High	Low	Low	Low	Low	High
Characteristics of admissions													
Total admissions	2462	2456	4110	1279	1833	3405	2262	2960	1506	2323	1782	1838	1825
Median age (IQR)	25 (12–48)	15 (8–31.5)	30 (13–60)	17 (9–32)	17 (9–32)	12 (7–24)	27 (13–54)	24 (11–60)	15 (8–36)	13 (7–26)	14 (7–36)	19 (10–36)	30 (15–60)
Males (%)	1337 (54)	1460 (59)	2136 (52)	675 (52)	1044 (57)	1916 (56)	1212 (54)	1548 (52)	863 (57)	1261 (54)	978 (55)	950 (52)	954 (52)
Inpatient mortality (%)	179 (7.3)	120 (4.9)	299 (7.3)	39 (3.0)	46 (2.5)	138 (4.1)	149 (6.6)	205 (6.9)	86 (5.7)	188 (8.1)	197 (11.1)	47 (2.6)	115 (6.3)
Mean WAZ (SD)	−1.0 (1.4)	−1.4 (1.6)	−0.9 (1.5)	−1.1 (1.6)	−1.2 (1.5)	−1.5 (1.6)	−0.9 (1.4)	−0.7 (1.6)	−1.3 (1.5)	−1.4 (1.7)	−1.6 (1.6)	−0.9 (1.6)	−0.8 (1.5)

WAZ, Weight for age z-score.

### Admission diagnoses

There were 55 705 distinct admission diagnoses among the 30 042 admissions and 17 710 patients (58.9%) had multiple diagnoses. The leading admission diagnoses are shown in table 2 stratified by patient age and hospitals grouped by malaria prevalence. Over 70% admissions to five hospitals in high malaria prevalence areas had malaria. Approximately one-third of under-fives in these hospitals were diagnosed with pneumonia and a similar percentage had diarrhoea/dehydration. In older children (≥60 months), anaemia (22%) and sickle cell disease (10%) featured among the common diagnoses. Within the eight low malaria prevalence hospitals, pneumonia (53%) and dehydration (35%) were the most common diagnoses. Among children older than 59 months, non-infectious diseases including rickets and convulsive disorders (including all epilepsies) became prominent. Cumulatively, the 10 leading diagnoses were present in between 80% and 97.2% of admissions depending on the malaria setting and patient age.

**Table 2:** Admission diagnosis in children admitted to Clinical Information Network hospitals according to age and malaria prevalence

High malaria prevalence (n=5 hospitals)				Low malaria prevalence (n=8 hospitals)			
2–59 months		60 months and above		2–59 months		60 months and above	
Diagnosis	Frequency (%)	Diagnosis	Frequency (%)	Diagnosis	Frequency (%)	Diagnosis	Frequency (%)
Malaria	7512/10 298 (72.95)	Malaria	2528/3287 (76.91)	Pneumonia	7760/14 584 (53.21)	Pneumonia	447/1794 (24.92)
Pneumonia	3524/10 298 (34.22)	Anaemia	723/3287 (22%)	Dehydration	5215/14 584 (35.76)	Meningitis	356/1794 (19.84)
Dehydration	3353/10 298 (32.56)	Dehydration	468/3287 (14.24)	Meningitis	1727/14 584 (11.84)	Dehydration	266/1794 (14.83)
Anaemia	1749/10 298 (16.98)	Pneumonia	465/3287 (14.15)	Malnutrition	1718/14 584 (11.78)	Malaria	224/1794 (12.49)
Meningitis	980/10 298 (9.52)	Meningitis	397/3287 (12.08)	Febrile convulsion	1274/14 584 (8.74)	Anaemia	169/1794 (9.42)
Malnutrition	573/10 298 (5.56)	Sickle cell disease	310/3287 (9.43)	Malaria	714/14 584 (4.9)	Convulsive disorder	169/1794 (9.42)
URTI	411/10 298 (3.99)	URTI	104/3287 (3.16)	Rickets	508/14 584 (3.48)	Asthma	114/1794 (6.35)
Sickle cell disease	267/10 298 (2.59)	Malnutrition	93/3287 (2.83)	Anaemia	471/14 584 (3.23)	Febrile convulsion	102/1794 (5.69)
Febrile convulsion	248/10 298 (2.41)	Poisoning	88/3287 (2.68)	Convulsive disorder	468/14 584 (3.21)	HIV	101/1794 (5.63)
Poisoning	173/10 298 (1.68)	HIV	84/3287 (2.56)	URTI	444/14 584 (3.04)	Poisoning	89/1794 (4.96)
Number of admissions with at least one of 10 leading diagnoses	10 010/10 298 (97.2)	Number of admissions with at least one of 10 leading diagnoses	3040/3287 (92.49)	Number of admissions with at least one of 10 leading diagnoses	13 278/14 584 (91.04)	Number of admissions with at least one of 10 leading diagnoses	1436/1794 (80.04)
Proportion of all diagnoses that are one of top 10 diagnoses	18 790/26 987 (69.63)	Proportion of all diagnoses that are one of top 10 diagnoses	5260/26 987 (19.49)	Proportion of all diagnoses that are one of top 10 diagnoses	20 299/28 689 (70.76)	Number of episodes contributing to top 10 diagnosis	2037/28 689 (7.1)

URTI, upper respiratory tract infection.

### Inpatient mortality

**Figure 1:**
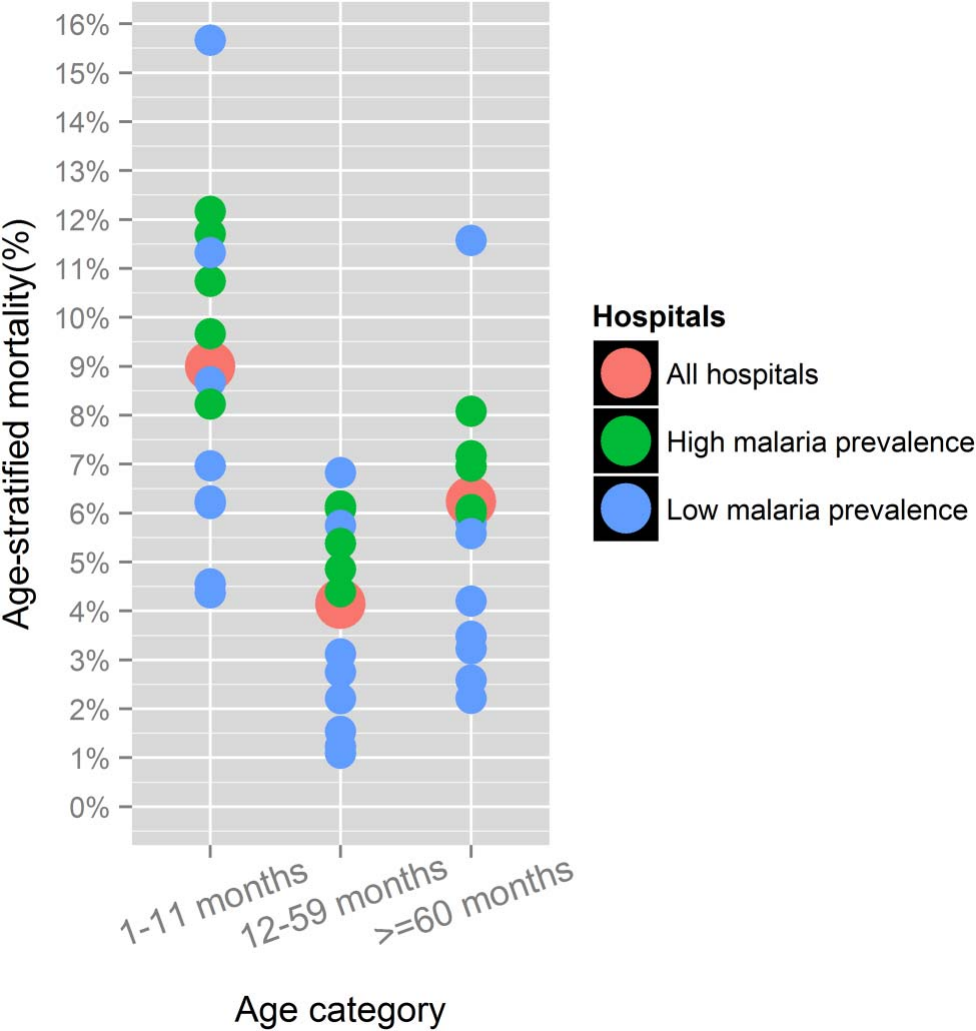
Inpatient mortality in children admitted to Kenyan hospitals stratified by age and malaria prevalence.

**Figure 2:**
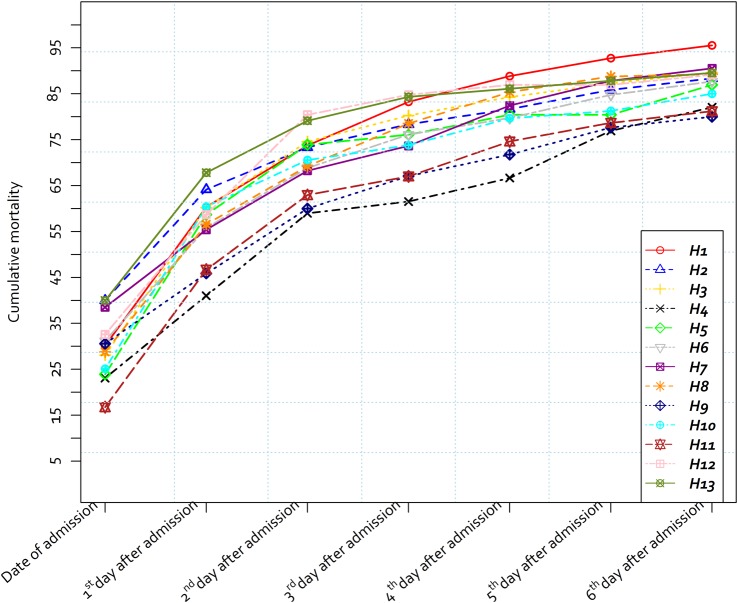
Inpatient mortality during the first week of admission in children admitted to Kenyan hospitals.

Overall, 1808 inpatient deaths (6%) occurred but rates varied substantially across hospitals (range 2.5%–11.1%) (table 1). Of all inpatient deaths, 535 (29.6%) occurred on the date of admission (range per hospital, 16.7%–40%), and 1037 deaths (57.4%) had occurred by or on day 2 (range 41%–67.8%, figure 1). Infants 1–11 months had consistently higher mortality rates in all hospitals but mortality for those aged over 5 years was also high (figure 2).

**Figure 3:**
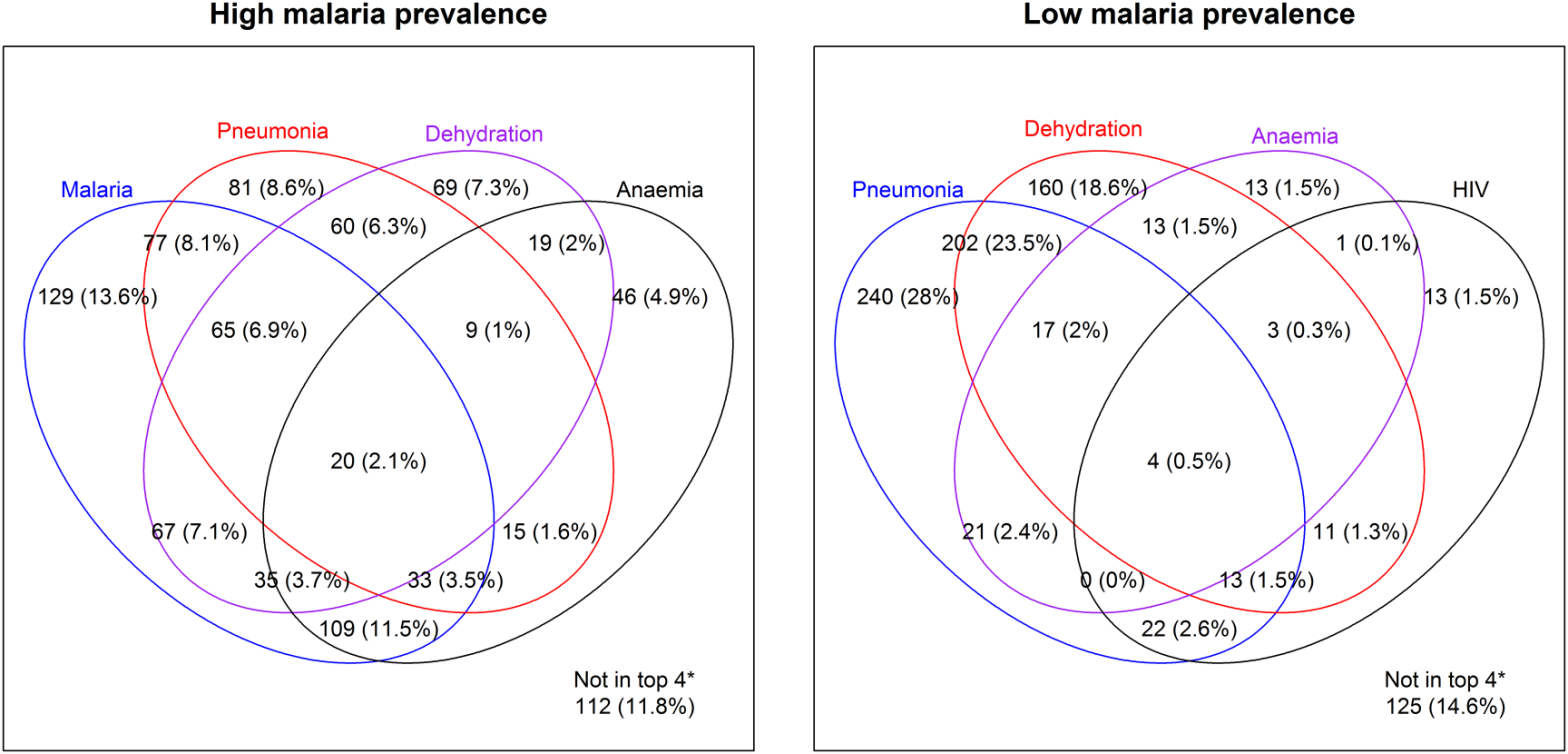
Causes of inpatient deaths after excluding meningitis and severe acute malnutrition. *Deaths within the ‘other’ category had none of the four leading diagnoses as a primary or comorbid condition.

Based on the assigned hierarchy of admission clinical diagnosis, the median case fatality rate for clinically diagnosed meningitis was 9% (range 3%–17%) and for severe malnutrition was 19% (range 6%–30%). Other deaths were predominantly associated with multi-morbidity (figure 3). At least 80% of inpatient deaths in each hospital that were not attributable to meningitis or severe malnutrition were related to four diagnoses: pneumonia, diarrhoea/ dehydration, anaemia and HIV or malaria (in low and high malaria prevalence areas, respectively).

### Investigation of common childhood illness

A summary of laboratory investigations in children presenting with common clinical syndromes: malaria, febrile illness (with or without clinical malaria), meningitis and severely ill admissions with any danger sign is presented in table 3 (a full description by hospital is available in online supplementary Webtable 1). Depending on the hospital, the diagnosis of clinical malaria was confirmed by positive laboratory findings (blood slide) for between 69% and 89% of children with clinical malaria in high prevalence hospitals (median=79%) and 5% and 100% of malaria admissions in low prevalence hospitals (median=65%). Up to 95% of malaria tests in children with a malaria diagnosis were negative (11%–31% in high prevalence (median=21%) and 0%–95% in low prevalence areas (median=35%)), and between 0% and 67% of results (median=16%) were not documented in laboratory or clinical records. Of the children admitted with severe pallor across hospitals, a median proportion of only 8% (range 1%–37%) had haemoglobin testing ordered and results were available for a median of 85% (range 50%–94%) of these haemoglobin investigations.

**Table 3:** Investigations conducted in paediatric admissions to Clinical Information Network hospitals

Indicator	Median percentage in H1–H13 (%)	Range (%)
HIV testing among all admissions		
HIV test ordered	34	2–64
HIV status ascertained	38	12–76
HIV status previously known	1	0–6
Children with danger signs	67	52–85
Blood glucose in children with danger signs	18	1–63
Total fever cases	80	71–88
Fever cases with malaria diagnosis	12	0–92
Urine test (dipstix, microbiology and culture) ordered among children with fever	3	1–14
Microbiology and culture test	3	0–24
Blood culture done among children with fever	0	0–5
Patients treated for malaria on admission	10	0–88
Percentage with negative malaria slides	32	0–100
Haemoglobin ordered for admissions with severe pallor	8	1–37
Haemoglobin results available for admissions with severe pallor	85	50–94
Pneumonia admissions	50	22–55
Chest X-ray ordered	9	4–31
Patients treated for meningitis on admission	13	3–21
Lumbar puncture ordered	66	38–95
Lumbar puncture result available	58	15–79

Among all children with febrile illness (n=18 693), the diagnostic investigations employed in identifying causes of fever other than malaria testing were urine testing (median=3%, range 1%–14%) and blood culture (0%, 0%–5%). In children with a clinical diagnosis of meningitis a median proportion of 66% (range 38%–95%) had lumbar puncture ordered, but in only 6 out of 10 cases (median 58%, range 15%–79%) were the findings of these lumbar punctures documented in laboratory or clinical records suggesting many lumbar punctures ordered are not done. The median proportion of children admitted to CIN hospitals who had HIV status ascertained (encompassing clearly documented maternal testing history or diagnostic testing in the child) at any point during the current hospitalisation was 38% (range 12%–76%).

Occurrence of at least one danger sign (central cyanosis, inability to drink/breastfeed, altered consciousness or grunting) suggesting severe illness was found in a median percentage of 67 admissions (range 52%–85%). Between 1% and 63% (median=18%) of these children with danger signs had a blood glucose test conducted within the initial hours of hospitalisation, as is recommended.

### Treatment of common illness

We evaluated prescription of antimalarial drugs, antibiotics and therapeutic feeding by hospital (table 4, a full description by hospital is available in online supplementary webtable 2). Ceftriaxone use for meningitis varied widely with a median proportion of 13% (0%–83%), while the majority of patients with meningitis received chloramphenicol and penicillin. Among the admissions with severe acute malnutrition, a median proportion of 65% (18%–87%) received guideline-recommended antibiotic cover (penicillin in combination with gentamicin). Therapeutic feeds (either F75, F100 or ready-to-use therapeutic feeds) were prescribed in a median proportion of 49.5% (9%–93%) of patients with severe acute malnutrition. After excluding patients with meningitis and/or severe acute malnutrition, a median proportion of 35% (12%–65%) patients with pneumonia received penicillin in combination with gentamicin, 33% (16%–60%) received penicillin alone and 2% (0%–32%) received amoxicillin. Considering all gentamicin prescriptions regardless of diagnosis for which gentamicin was prescribed, overdosing was infrequent but consistent across hospitals (median 2%, range 0%–5%) with occasional prescription of very high (toxic) doses (median 1%, range 0%–2%). Antimalarial treatment of severe malaria varied widely among hospitals (quinine (range 0%–75%) and artesunate (0%–79%)).

**Table 4:** Treatment of common childhood illnesses in admissions to Clinical Information Network hospitals

Indicator	Median per cent in H1-H13 (%)	Range (%)
Clinician's diagnosis of malaria	10	0–88
Proportion treated with artesunate	25	0–79
Proportion treated with quinine	2	0–75
Proportion starting treatment on oral ACT	6	1–50
Clinician’s diagnosis of meningitis	13	3–21
Proportion treated with penicillin and chloramphenicol	51	0–86
Proportion treated with ceftriaxone alone	12	0–83
Clinician’s diagnosis of severe acute malnutrition (not meningitis)	6	3–17
Proportion treated with F75 or F100 or RUTF	59	9–130
Proportion treated with penicillin and gentamicin	65	18–87
Pneumonia (excluding meningitis and severe acute malnutrition)	41	20–48
Proportion treated with penicillin and gentamicin	40	12–65
Proportion treated with penicillin alone	33	16–60
Proportion starting treatment with amoxicillin	2	0–32
Clinician’s diagnosis of diarrhoea/dehydration	30	19–43
Proportion treated with zinc	71	27–89
Gentamicin prescriptions	20	9–55
Gentamicin overdose (>20% recommended dose)	2	0–5
Gentamicin overdose (>50% recommended dose)	1	0–2

ACT – artemisinin based combination therapy RUTF, ready-to-use therapeutic feeds.

## Discussion

In this descriptive report, we present data for the first complete year of network activities in 13 hospitals. In common with other reports from our setting, presentation with multiple admission diagnoses is common.^14^ Leading causes of admission vary with the patient age and malaria prevalence. The high proportion of children admitted with pneumonia is worthy of note despite more than 10 years use of HiB vaccine and 2 years use of PCV10 vaccine. Clinical or confirmed HIV diagnoses were rarely made, while diagnoses like sickle cell disease and rickets that receive little policy attention feature among leading diagnoses in older children in some hospitals.

The findings of inpatient mortality analysis confirm that most deaths are associated with common diagnoses and multi-morbidity and these deaths commonly occur within the first 48 h of admission.^15^ Substantial variability in mortality across hospitals is observed but a pattern of higher mortality in infants was consistent. Although admissions of children aged over 5 years are less common, mortality in this group was high. Further work to explore variation in mortality and conduct systematic audit of deaths are warranted and can now be tackled within the CIN. These findings support efforts to ensure health workers can perform effective triage and emergency care and then manage common childhood admissions.^11^ ^12^ The findings also suggest a continued need for improved early referral from primary care settings. Almost two decades after the launch globally of the Integrated Management of Childhood Illnesses strategy and the more recent launch of Integrated Community Case Management, it appears that referral systems remain weak. Data from the CIN may also help focus attention on these weaknesses.

The decision to perform diagnostic tests during admission differed widely from hospital to hospital and by illness. Even for illnesses where investigations are commonly ordered such as malaria, the results are not always available and negative results do not always inform care. Haemoglobin for severe clinical pallor and blood glucose for severe illness are often not checked although the risk of death is related to absolute levels of haemoglobin,^16^ and hypoglycaemia may be common and associated with death.^17^ Results for haemoglobin and blood glucose measurement and lumbar puncture were also often missing even from laboratories and suggest that even when ordered many tests are not actually done. Although clear recommendations exist to offer routine HIV testing this was still low even in hospitals in areas with high maternal antenatal HIV prevalence. Limited use of the diagnostic tests and cross-site variability are largely related to poor availability of equipment and reagents or related to costs which families who often pay for diagnostic tests cannot afford in our experience. This also seems to explain the fact that clinicians rarely investigate for other causes of fever than malaria. Blood cultures may be an important source of information to determine aetiology of bacterial infection (also useful for surveillance) and may guide treatment.^18^ ^19^ However, most hospitals cannot offer blood culture routinely and use of urine culture is extremely low. Despite high mortality, hospitals remain poorly equipped to provide even a basic panel of investigations for sick children in an era when universal coverage with quality care is being espoused.

There were areas with notable improvements in treatment of common childhood illnesses compared with previous reports in Kenyan facilities.^20^ These areas include zinc supplementation in diarrhoea, and prescription of therapeutic feeds for severe acute malnutrition, although both still need to be improved. Our analyses also reveal areas requiring further improvement. Three years after a national policy change to artesunate to treat severe malaria, which may reduce mortality by 20% compared with quinine,^21^ fewer than half of cases are receiving this drug although this too is highly variable across place. Second, antibiotic prescription errors, specifically overdosing, occur in up to 4% of children who are prescribed gentamicin and, in common with previous reports, in about one-half of these cases the prescribed dose was very high.^20^

The CIN aims to provide data on the routine care provided in Kenyan hospitals. Its reliance on documentation in clinical records to determine what care is provided is a limitation, but remains the only realistic way to capture such data at large scale. To mitigate this limitation considerable efforts to improve data quality are made including follow-up in the laboratory to confirm whether there was evidence that investigations were performed and their results. There are also clear limitations in assigning diagnoses in the absence of many diagnostic tests. It may be more appropriate therefore to consider many diagnoses as clinical syndromes rather than pathological entities. Despite falls in child mortality in Kenya^22^ and introduction of vaccines to prevent meningitis and pneumonia, common largely infectious illnesses still account for most admissions and are associated with most deaths with infants remaining particularly vulnerable.

In conclusion, CIN set out to provide a new platform to help characterise hospital care in detail. As the era of the Millennium Development Goals ends it has been able to demonstrate that it is possible to collect data at reasonable scale. The data demonstrate that hospital mortality remains high in Kenya, resources to investigate and treat severe illness remain limited, care provided and outcomes vary widely across hospitals and adoption of effective interventions remains slow. Going forward CIN aims to support efforts to improve care and reduce variation in practice, shortening the time from knowledge acquisition to patient impact, engage hospitals providing routine care in research on how best to make improvements and to test effects of interventions in routine settings, increasing the availability of the much-needed pragmatic research.^23^ ^24^

## Supplementary Material

Web table 1

Web table 2
