# Supplementary material for: Characteristics of admissions and variations in the use of basic investigations, treatments and outcomes in Kenyan hospitals within a new Clinical Information Network
Source: Arch Dis Child. 2015 Dec 10;101(3):223–9. doi: 10.1136/archdischild-2015-309269 (PMC4789757; doi:10.1136/archdischild-2015-309269)
Supplement: Web table 1 [file archdischild-2015-309269-s1.pdf]

Webtable 1: Investigations conducted in admissions to CIN hospitals

**Indicator**	**H1**	**H2**
HIV test ordered	898/2188 (41%)	1409/2193 (64%)
HIV Status ascertained	858/2188 (39%)	1355/2193 (62%)
HIV status previoulsy known	12/858 (1%)	0/1355 (0%)
**Children with danger signs***	**1710/2188 (78%)**	**1309/2193 (60%)**
Blood glucose in children with danger signs	36/1710 (2%)	438/1309 (33%)
**Total fever cases**	**1922/2188 (88%)**	**1607/2193 (73%)**
Fever cases with malaria diagnosis	1512/1922 (79%)	190/1607 (12%)
Urine test ordered among children with fever	72/1922 (4%)	22/1607 (1%)
Microbilogy and Culture test	4/72 (6%)	1/22 (5%)
Blood culture done among children with fever	3/1922 (0%)	10/1607 (1%)
**Patients treated for malaria on admission**	**1588/2188 (73%)**	**209/2193 (10%)**
Percentage with negative malaria slides	327/1504 (22%)	105/142 (74%)
**Hemoglobin ordered for admissions with severe pallor**	**323/1197(27%)**	**24/797(3%)**
Hemoglobin results available for admissions with severe pallor	272/323(84%)	20/24(83%)
**Pneumonia admissions**	**654/2188 (30%)**	**1199/2193 (55%)**
Chest xray ordered	91/654 (14%)	111/1199 (9%)
**Patients treated for meningitis on admission**		
Lumbar Puncture ordered	54/68 (79%)	263/354 (74%)
Lumbar Puncture result available	21/54 (39%)	151/263 (57%)

H3	H4	H5	H6	H7
81/3659 (2%)	230/1159 (20%)	400/1590 (25%)	109/1041 (10%)	893/1860 (48%)
445/3659 (12%)	223/1159 (19%)	295/1590 (19%)	304/1041 (29%)	856/1860 (46%)
16/445 (4%)	1/223 (0%)	0/295 (0%)	2/304 (1%)	1/856 (0%)
**3099/3659 (85%)**	**721/1159 (62%)**	**1067/1590 (67%)**	**555/1041 (53%)**	**1217/1860 (65%)**
36/3099 (1%)	198/721 (27%)	189/1067 (18%)	39/555 (7%)	54/1217 (4%)
**3097/3659 (85%)**	**862/1159 (74%)**	**1136/1590 (71%)**	**829/1041 (80%)**	**1533/1860 (82%)**
2837/3097 (92%)	7/862 (1%)	79/1136 (7%)	2/829 (0%)	1073/1533 (70%)
20/3097 (1%)	10/862 (1%)	67/1136 (6%)	9/829 (1%)	65/1533 (4%)
0/20 (0%)	0/10 (0%)	0/67 (0%)	0/9 (0%)	3/65 (5%)
0/3097 (0%)	0/862 (0%)	0/1136 (0%)	1/829 (0%)	4/1533 (0%)
**3204/3659 (88%)**	**8/1159 (1%)**	**88/1590 (6%)**	**4/1041 (0%)**	**1189/1860 (64%)**
296/1808 (16%)	2/6 (33%)	20/21 (95%)	0/4 (0%)	199/955 (21%)
**423/1130(37%)**	**7/386(2%)**	**8/917(1%)**	**8/70(11%)**	**177/965(18%)**
322/423(76%)	6/7(86%)	4/8(50%)	6/8(75%)	156/177(88%)
**937/3659 (26%)**	**629/1159 (54%)**	**837/1590 (53%)**	**530/1041 (51%)**	**440/1860 (24%)**
64/937 (7%)	30/629 (5%)	100/837 (12%)	24/530 (5%)	40/440 (9%)
464/782 (59%)	123/197 (62%)	130/169 (77%)	49/83 (59%)	20/52 (38%)
361/464 (78%)	61/123 (50%)	70/130 (54%)	21/49 (43%)	3/20 (15%)

H8	H9	H10	H11	H12
832/2461 (34%)	833/1338 (62%)	219/1049 (21%)	685/1584 (43%)	986/1661 (59%)
856/2461 (35%)	1020/1338 (76%)	221/1049 (21%)	779/1584 (49%)	953/1661 (57%)
29/856 (3%)	14/1020 (1%)	0/221 (0%)	49/779 (6%)	2/953 (0%)
**1648/2461 (67%)**	**932/1338 (70%)**	**802/1049 (76%)**	**1302/1584 (82%)**	**856/1661 (52%)**
109/1648 (7%)	299/932 (32%)	260/802 (32%)	417/1302 (32%)	538/856 (63%)
**2052/2461 (83%)**	**975/1338 (73%)**	**828/1049 (79%)**	**1269/1584 (80%)**	**1213/1661 (73%)**
1510/2052 (74%)	46/975 (5%)	91/828 (11%)	156/1269 (12%)	14/1213 (1%)
53/2052 (3%)	111/975 (11%)	10/828 (1%)	178/1269 (14%)	51/1213 (4%)
3/53 (6%)	27/111 (24%)	0/10 (0%)	5/178 (3%)	0/51 (0%)
2/2052 (0%)	49/975 (5%)	0/828 (0%)	50/1269 (4%)	10/1213 (1%)
**1677/2461 (68%)**	**58/1338 (4%)**	**100/1049 (10%)**	**172/1584 (11%)**	**14/1661 (1%)**
478/1560 (31%)	31/45 (69%)	30/81 (37%)	33/104 (32%)	4/13 (31%)
**115/781(15%)**	**27/671(4%)**	**15/949(2%)**	**109/1290(8%)**	**8/284(3%)**
100/115(87%)	23/27(85%)	13/15(87%)	86/109(79%)	7/8(88%)
**991/2461 (40%)**	**673/1338 (50%)**	**548/1049 (52%)**	**863/1584 (54%)**	**728/1661 (44%)**
40/991 (4%)	102/673 (15%)	34/548 (6%)	266/863 (31%)	113/728 (16%)
54/92 (59%)	165/188 (88%)	89/212 (42%)	190/201 (95%)	45/55 (82%)
36/54 (67%)	107/165 (65%)	68/89 (76%)	110/190 (58%)	27/45 (60%)

H13	Median	Range
524/1563 (34%)	34%	(2,64)
601/1563 (38%)	38%	(12,76)
4/601 (1%)	1%	(0,6)
**1279/1563 (82%)**	**67%**	**(52,85)**
36/1279 (3%)	18%	(1,63)
**1370/1563 (88%)**	**80%**	**(71,88)**
1040/1370 (76%)	12%	(0,92)
32/1370 (2%)	3%	(1,14)
1/32 (3%)	3%	(0,24)
0/1370 (0%)	0%	(0,5)
**1108/1563 (71%)**	**10%**	**(0,88)**
99/881 (11%)	31%	(0,95)
**83/319(26%)**	**8%**	**(1,37)**
78/83(94%)	85%	(50,94)
**344/1563 (22%)**	**50%**	**(22,55)**
29/344 (8%)	9%	(4,31)
144/217 (66%)	66%	(38,95)
114/144 (79%)	58%	(15,79)
