# Supplementary material for: Characteristics of admissions and variations in the use of basic investigations, treatments and outcomes in Kenyan hospitals within a new Clinical Information Network
Source: Arch Dis Child. 2015 Dec 10;101(3):223–9. doi: 10.1136/archdischild-2015-309269 (PMC4789757; doi:10.1136/archdischild-2015-309269)
Supplement: Web table 2 [file archdischild-2015-309269-s2.pdf]

Webtable 2: Treatment of common childhood illnesses in admissions to CIN hospitals

**Indicator**	**H1**
**Clinician's diagnosis of malaria**	**1588/2188 (73%)**
Proportion treated with Artesunate	404/1588 (25%)
Proportion treated with quinine	1079/1588 (68%)
Proportion starting treatment on oral ACT	56/1588 (4%)
**Clinician's diagnosis of meningitis**	**68/2188 (3%)**
Proportion treated with Penicillin and Chloramphenicol	0/68 (0%)
Proportion treated with Ceftriaxone alone	53/68 (78%)
**Clinician's diagnosis of Severe Acute Malnutrition (not meningitis)**	**127/2188 (6%)**
Proportion treated with F75 or F100 or RUTF	102/127 (80%)
Proportion treated with Penicillin & Gentamicin	110/127 (87%)
**Pneumonia (excluding meningitis & severe acute malnutrition)**	**598/2188 (27%)**
Proportion treated with Penicillin & Gentamicin	207/598 (35%)
Proportion treated with Penicillin alone	309/598 (52%)
Proportion starting treated with Amoxicillin	42/598 (7%)
**Clinician's diagnosis of diarrhoea / dehydration**	**519/2188 (24%)**
Proportion treated with Zinc	401/519 (77%)

H2	H3	H4	H5	H6
**209/2193 (10%)**	**3204/3659 (88%)**	**8/1159 (1%)**	**88/1590 (6%)**	**4/1041 (0%)**
62/209 (30%)	964/3204 (30%)	1/8 (12%)	0/88 (0%)	2/4 (50%)
3/209 (1%)	2020/3204 (63%)	2/8 (25%)	3/88 (3%)	0/4 (0%)
14/208 (7%)	39/3203 (1%)	1/8 (12%)	1/87 (1%)	2/4 (50%)
**354/2193 (16%)**	**782/3659 (21%)**	**197/1159 (17%)**	**169/1590 (11%)**	**83/1041 (8%)**
247/354 (70%)	4/782 (1%)	142/197 (72%)	95/169 (56%)	71/83 (86%)
30/354 (8%)	651/782 (83%)	18/197 (9%)	22/169 (13%)	0/83 (0%)
**359/2193 (16%)**	**121/3659 (3%)**	**72/1159 (6%)**	**80/1590 (5%)**	**127/1041 (12%)**
213/359 (59%)	48/121 (40%)	18/72 (25%)	104/80 (130%)	118/127 (93%)
211/359 (59%)	64/121 (53%)	30/72 (42%)	58/80 (72%)	98/127 (77%)
**896/2193 (41%)**	**799/3659 (22%)**	**538/1159 (46%)**	**767/1590 (48%)**	**444/1041 (43%)**
175/896 (20%)	259/799 (32%)	148/538 (28%)	380/767 (50%)	153/444 (34%)
463/896 (52%)	268/799 (34%)	268/538 (50%)	246/767 (32%)	268/444 (60%)
33/896 (4%)	110/799 (14%)	7/538 (1%)	7/767 (1%)	2/444 (0%)
**724/2193 (33%)**	**1066/3659 (29%)**	**379/1159 (33%)**	**392/1590 (25%)**	**447/1041 (43%)**
520/724 (72%)	635/1066 (60%)	237/379 (63%)	213/392 (54%)	396/447 (89%)

H7	H8	H9	H10	H11
**1189/1860 (64%)**	**1677/2461 (68%)**	**58/1338 (4%)**	**100/1049 (10%)**	**172/1584 (11%)**
125/1189 (11%)	1317/1677 (79%)	2/58 (3%)	70/100 (70%)	130/172 (76%)
896/1189 (75%)	25/1677 (1%)	6/58 (10%)	2/100 (2%)	0/172 (0%)
19/1188 (2%)	152/1676 (9%)	15/57 (26%)	1/100 (1%)	3/172 (2%)
**52/1860 (3%)**	**92/2461 (4%)**	**188/1338 (14%)**	**212/1049 (20%)**	**201/1584 (13%)**
8/52 (15%)	68/92 (74%)	15/188 (8%)	92/212 (43%)	103/201 (51%)
10/52 (19%)	7/92 (8%)	85/188 (45%)	44/212 (21%)	11/201 (5%)
**84/1860 (5%)**	**162/2461 (7%)**	**77/1338 (6%)**	**75/1049 (7%)**	**275/1584 (17%)**
24/84 (29%)	141/162 (87%)	64/77 (83%)	28/75 (37%)	47/275 (17%)
37/84 (44%)	103/162 (64%)	50/77 (65%)	60/75 (80%)	203/275 (74%)
**417/1860 (22%)**	**898/2461 (36%)**	**578/1338 (43%)**	**464/1049 (44%)**	**693/1584 (44%)**
167/417 (40%)	559/898 (62%)	296/578 (51%)	247/464 (53%)	450/693 (65%)
100/417 (24%)	256/898 (29%)	202/578 (35%)	128/464 (28%)	108/693 (16%)
7/417 (2%)	21/898 (2%)	6/578 (1%)	6/464 (1%)	10/693 (1%)
**526/1860 (28%)**	**939/2461 (38%)**	**404/1338 (30%)**	**397/1049 (38%)**	**549/1584 (35%)**
405/526 (77%)	772/939 (82%)	288/404 (71%)	280/397 (71%)	150/549 (27%)

H12	H13	Median	Range
**14/1661 (1%)**	**1108/1563 (71%)**	10%	(0,88)
0/14 (0%)	212/1108 (19%)	25%	(0,79)
0/14 (0%)	763/1108 (69%)	2%	(0,75)
4/13 (31%)	68/1099 (6%)	6%	(1,50)
**55/1661 (3%)**	**217/1563 (14%)**	13%	(3,21)
11/55 (20%)	152/217 (70%)	51%	(0,86)
23/55 (42%)	10/217 (5%)	12%	(0,83)
**74/1661 (4%)**	**56/1563 (4%)**	6%	(3,17)
7/74 (9%)	34/56 (61%)	59%	-9,130
13/74 (18%)	46/56 (82%)	65%	(18,87)
**679/1661 (41%)**	**310/1563 (20%)**	41%	(20,48)
82/679 (12%)	148/310 (48%)	40%	(12,65)
222/679 (33%)	99/310 (32%)	33%	(16,60)
219/679 (32%)	43/310 (14%)	2%	(0,32)
**496/1661 (30%)**	**302/1563 (19%)**	30%	(19,43)
281/496 (57%)	228/302 (75%)	71%	(27,89)
